# Frontiers in the Expansion of Bioproducts

**DOI:** 10.1155/2016/3809386

**Published:** 2016-10-31

**Authors:** Junio Cota, Joachim Venus, Zaira B. Hoffmam, Lucas F. Ribeiro

**Affiliations:** ^1^Institute of Agricultural Sciences (ICA), Universidade Federal de Minas Gerais (UFMG), Montes Claros, MG, Brazil; ^2^Department of Bioengineering, Leibniz Institute for Agricultural Engineering, Bornim, Potsdam, Germany; ^3^Brazilian Bioethanol Science and Technology Laboratory (CTBE), Campinas, SP, Brazil; ^4^Department of Chemical and Biomolecular Engineering, Johns Hopkins University, Baltimore, MD, USA

Bioproducts and biobased technologies are increasingly taking on outstanding position in the global market. Although there is debate on the longevity of the oil reserves, the global warming and its devastating consequences cannot be denied and probably will put an end to the fossil fuels era and start a new one with dependence on renewable energy. In December 2015, in an unprecedented agreement, 195 nations (including all of the top 10 emitting countries) committed to The Paris Agreement to hold “the increase in the global average temperature to well below 2°C above preindustrial levels and to pursue efforts to limit the temperature increase to 1.5°C above pre-industrial levels, recognizing that this would significantly reduce the risks and impacts of climate change” [1], which presumably would promote a significant increase in investments in green technologies.

Renewable raw materials have already been used to manufacture a wide variety of bioproducts in biorefineries worldwide, which may be divided into three large groups: biochemicals, biomaterials, and energy fuels. The concept of a self-sustainable biorefinery would ideally share manufacturing processes to produce bulk chemicals and high market value biobased chemicals at low cost, using cheap substrate and/or processes. However, industrial processes are mostly far from that model, since a lot of bottlenecks arise from each particular bioprocess. Nevertheless, 50 million tonnes of bioproducts were generated in 2012, including nonfood starch, cellulose fibres and cellulose derivatives, tall oils, fatty acids, and fermentation products such as alcohols, ketones, esters, organic acids, biopolymers, enzymes, amino acids, and vitamins [2]. Currently, the production of biobased commodity is commonly impaired by the competitive low price of crude oil, which constitutes a barrier to justify production costs. In a broader perspective, worldwide exportations of products in agriculture and forestry, food, bioenergy, biotechnology, and green chemistry were estimated to be about US $2 trillion in 2014, accounting for 13% of world trade [3]. The global renewable chemicals market reached a remarkable size of US $49 billion in 2015 and is forecasted to increase to US $84.3 billion by 2020. Just the global market for fermentation derived fine chemicals saw a revenue of US $16 billion in 2009, this sector being boosted by modern biotechnology that allows industry to improve the economics of new and old fermentation products.

In this context, this special issue introduces new concepts and trends regarding biobased processes and consists of four original researches and one review article. In one article, I. Baumann and P. Westermann review the current technologies and market aspects for short chain fatty acids synthesis by anaerobic fermentation of nonfood biomass, with a focus on a sustainable industrial production. In the first research article, X. Zhu et al. report enzymatic synthesis of precursors of important steroid drugs by biotransformation of cholesterol using* Burkholderia cepacia* strains under different nutritional conditions. In another research article, K. Godlewska et al. report the effects of bioproducts extracted from seaweed on agriculture/horticulture, which comprises increment in plant height and assimilation of microelements and in chlorophyll content. In a third research article, Y. Li et al. report synthesis of pyrimethanil grafted chitosan derivatives with enhanced antifungal activity against plant pathogenic fungi in comparison with chitosan. Finally, Z. Zahan et al. report the effects of anaerobic codigestion of wastes from food manufacturing and processing companies using municipal wastewater treatment plant's primary sludge and waste activated sludge, and their research suggests that codigestion has great potential in improving the specific biogas production and methane yield with sewage sludge.

In summary and given the importance of raising bioeconomy, the present issue aims to expand our comprehension of different bioprocesses for synthesis of biobased products with economical relevance. In this sense, more than 40 nations are already in the way of establishing a consolidated bioeconomy, which will be a result of some initiatives such as close association of multilateral policy processes and intergovernmental discussions, international collaborations between governments and public and private researchers, international collaboration between researchers to evolve and disseminate the knowledge, and establishment of R&D support programmes [3]. The present issue brings together new potential technologies and methods to lead to an improvement of bioproducts manufacturing worldwide. While meeting those objectives, the work will also provide valuable source of reference for students and researchers.

*Junio Cota* *Joachim Venus* *Zaira B. Hoffmam* *Lucas F. Ribeiro*

## References

[1] Schellnhuber H. J., Rahmstorf S., Winkelmann R. (2016). Why the right climate target was agreed in Paris. *Nature Climate Change*.

[2] De Jong E., Higson A., Walsh P., Wellisch M. Bio-based chemicals—value added products from biorefineries. http://www.ieabioenergy.com/.

[3] El-Chichakli B., von Braun J., Lang C., Barben D., Philp J. (2016). Policy: five cornerstones of a global bioeconomy. *Nature*.

